# The STAT3-Ser/Hes3 signaling axis: an emerging regulator of endogenous regeneration and cancer growth

**DOI:** 10.3389/fphys.2013.00273

**Published:** 2013-10-01

**Authors:** Steven W. Poser, Deric M. Park, Andreas Androutsellis-Theotokis

**Affiliations:** ^1^Department of Medicine, University of DresdenDresden, Germany; ^2^Department of Neurological Surgery, University of VirginiaVA, USA; ^3^Center for Regenerative Therapies DresdenDresden, Germany

**Keywords:** stem cells, regenerative medicine, Hes3, STAT3 transcription factor, signal transduction

## Abstract

Stem cells, by definition, are able to both self-renew (give rise to more cells of their own kind) and demonstrate multipotential (the ability to differentiate into multiple cell types). To accommodate this unique dual ability, stem cells interpret signal transduction pathways in specialized ways. Notable examples include canonical and non-canonical branches of the Notch signaling pathway, with each controlling different downstream targets (e.g., Hes1 vs. Hes3) and promoting either differentiation or self-renewal. Similarly, stem cells utilize STAT3 signaling uniquely. Most mature cells studied thus far rely on tyrosine phosphorylation (STAT3-Tyr) to promote survival and growth; in contrast, STAT3-Tyr induces the differentiation of neural stem cells (NSCs). NSCs use an alternative phosphorylation site, STAT3-Ser, to regulate survival and growth, a site that is largely redundant for this function in most other cell types. STAT3-Ser regulates Hes3, and together they form a convergence point for several signals, including Notch, Tie2, and insulin receptor activation. Disregulation and manipulation of the STAT3-Ser/Hes3 signaling pathway is important in both tumorigenesis and regenerative medicine, and worthy of extensive study.

## Encoding multiple functions within the common signalosome

Stem cells exhibit the fundamental properties of both self-renewal and differentiation into multiple cell types. The balance between these two must be finely regulated in order to allow stem cells to properly contribute to tissue formation during development and later, in the context of resisting degenerative disease and normal wear-and-tear as we age. Key to this balance is the clear allocation of these two functions to distinct signal transduction pathways. Nature had two options to accomplish this task: (a) to put aside part of the signaling machinery to be used exclusively for self-renewal while have another totally separate set for regulating their multipotential nature, or, (b) to have individual cell types interpret the same signaling pathways differently. In the latter case, a stem cell could, for example, co-opt some typically pro-survival pathways, and utilize them as pro-differentiation signals. Consequently, a stem cell would have fewer pro-survival choices, but it would also be able to encode its multipotential nature without the need for possessing a unique set of signaling molecules. A specific example highlighting the second option is a signal transduction pathway that is emerging as a convergence point for diverse signals and regulates self-renewal vs. differentiation decisions with consequence in regenerative medicine, cancer biology, and drug discovery strategies. At the center of this pathway is the phosphorylation of Signal Transducer and Activator of Transcription 3 (STAT3) on the serine residue at amino acid position 727 (STAT3-Ser) and subsequent induction of the transcription factor Hairy and Enhancer of Split 3 (Hes3), a member of the Hes/Hey gene family of basic helix-loop-helix (bHLH) transcription factors.

## The neglected STAT3-Ser

The Janus kinase (JAK)-STAT pathway is a major, evolutionarily conserved signaling component in the cells of many organisms, ranging from slime molds to humans. Like other signal transduction cascades, its role is to convey information from the cell's environment all the way to DNA promoters on the genome, resulting in changes in gene expression. Binding of ligands, such as cytokines, hormones and growth factors to surface receptors leads to activation of JAKs. JAK proteins are a family of non-receptor tyrosine kinases and their most prominent targets are STAT proteins. Tyrosine-phosphorylated STATs then enter the nucleus where they regulate gene transcription. The long evolutionary history of the JAK-STAT pathway has incorporated it into many other signaling pathways making it relevant to a vast array of intrinsic and environmental stimuli, cell proliferation, development, hematopoiesis, immunity, metabolic regulation, and cancer (Levy and Darnell, 2002; Shen et al., 2004; Richard and Stephens, 2011; Vera et al., 2011; Wauman and Tavernier, 2011; Camporeale and Poli, 2012; Lafave and Levine, 2012; Mohr et al., 2012; O'shea and Plenge, 2012; Stark and Darnell, 2012; Tibes et al., 2012; Swiatek-Machado and Kaminska, 2013).

In addition to the tyrosine phosphorylation site, some STAT proteins also have a serine phosphorylation site (Decker and Kovarik, 2000; Reich, 2009). STAT-Ser phosphorylation is regulated by different pathways among STAT family members, as demonstrated by pharmacological approaches using receptor ligands and inhibitors of intracellular kinase cascades. In the case of STAT3, serine phosphorylation is induced by EGF, PDGF, insulin, IL-2, BCR, and TCR. In all these cases, STAT3-Ser phosphorylation is reduced by treatment with a MEK1/2 inhibitor (PD98059). In contrast, when STAT3-Ser phosphorylation is induced by treatment with IL-6 or IFN-alpha, phosphorylation is sensitive to a broad spectrum kinase inhibitor, H7 (Chung et al., 1997; Ng and Cantrell, 1997; Stephens et al., 1998; Su et al., 1999; Decker and Kovarik, 2000). Adding to the complexity of STAT-Ser phosphorylation regulation, multiple pathways activated by the same receptor impinge upon different STAT-Ser phosphorylations, adding to the complexity, as well as the utility, of STAT3 in regulating discrete biological processes (Kovarik et al., 1999; Lim and Cao, 1999; Decker and Kovarik, 2000). It will be highly informative to extend such studies using cells from different origins, and address how the regulation of STAT-Ser phosphorylation is affected by cell type, providing an additional level of specificity in terms of pharmacologically manipulating this site.

Early studies demonstrated that the role of STAT-Ser phosphorylation is largely auxiliary to the function of tyrosine-phosphorylated STAT3, resulting in an increase of its transcriptional activity (Levy and Darnell, 2002). More recent studies have uncovered potentially STAT-Ser—specific effects on cell growth. In this article we will focus on STAT3 because it is extensively studied in terms of its role in regulating the self-renewal and differentiation of neural stem cells (NSCs). In these cells, JAK2 phosphorylates STAT3-Tyr in response to the activation of several receptors, including the CNTF receptor. Tyrosine-phosphorylated STAT3 directly stimulates transcription of genes expressed in astrocytes, most notably glial GFAP, thereby promoting loss of the self-renewal state and differentiation toward the astroglial fate (Bonni et al., 1997; Rajan and McKay, 1998; Panchision and McKay, 2002). STAT3-Tyr phosphorylation is undetectable during self-renewal, showcasing its powerful role in inducing differentiation. This prompted the question of whether STAT3 is irrelevant to NSC growth, or whether NSCs utilized STAT3-Ser for regulating proliferation. Pharmacological and genetic (STAT3 phosphomimetic) approaches demonstrate that STAT3-Ser, in the absence of STAT3-Tyr, is a potent stimulator of NSC survival and growth (Androutsellis-Theotokis et al., 2006).

Many reports today implicate STAT3-Ser in cell growth, proliferation, and survival, in the context of cancer (Plaza-Menacho et al., 2007; Qin et al., 2008; Aggarwal et al., 2009; Lacreusette et al., 2009; Yeh et al., 2009; Banerjee et al., 2010; Sekine et al., 2011; Villalva et al., 2011; Miyakoshi et al., 2012; Tkach et al., 2013; Yang et al., 2013), insulin and neurotrophin signaling (Ng et al., 2006; Kim et al., 2009), neurite outgrowth (Zhou and Too, 2011), and hypertension (Zouein et al., 2013). An extensively studied aspect of STAT3 that is dependent upon serine phosphorylation is its involvement in the regulation of cellular respiration by localizing to the mitochondrion where it regulates reactive oxygen species production and cell death (Zhang et al., 2003; Shulga and Pastorino, 2012).

In this article, we address a particular branch of the signaling pathway involving STAT3-Ser and its downstream mediator, the transcription factor Hes3. This STAT3-Ser/Hes3 Signaling Axis does not operate in parallel to JAK-STAT, nor is it an auxiliary to it. In fact, STAT3-Tyr and STAT3-Ser represent distinct and opposing functions with significant consequences for cellular function and fate determination. Here we discuss its involvement in neuro-regenerative medicine as well as open questions that need to be addressed in order to elevate our understanding of this pathway toward therapeutic benefit.

## Hes3: A specialized member of the hes/hey gene family

The Hes/Hey family of bHLH transcription factors is a major target of the Notch signaling pathway (Artavanis-Tsakonas et al., 1999; Imayoshi and Kageyama, 2011; Ueo et al., 2012). They are expressed in NSCs and progenitor cells *in vitro* and *in vivo*, where they repress the expression of genes involved in differentiation, making them critical mediators of fate choice (Sasai et al., 1992; Lobe, 1997; Allen and Lobe, 1999; Hirata et al., 2001; Hatakeyama et al., 2004; Androutsellis-Theotokis et al., 2006, 2008a,b, 2009, 2010a,b; Basak and Taylor, 2007, 2009; Rueger et al., 2010; Imayoshi and Kageyama, 2011; Masjkur et al., 2012; Pacioni et al., 2012). Different experimental systems seem to regulate which Hes/Hey family members are more prominently expressed or modulated. For example, Hes1 opposes neuronal differentiation and therefore may support the maintenance of NSCs during mouse development (Hatakeyama et al., 2004; Imayoshi and Kageyama, 2011; Shimojo et al., 2011; Tan et al., 2012). Hes5 is expressed in multipotent progenitors in the embryonic neural tube as well as in neurospheres generated from this region *in vitro* (Hatakeyama et al., 2004; Basak and Taylor, 2007). In monolayer cultures of fetal and adult mouse NSCs, all family members are expressed (Androutsellis-Theotokis et al., 2006). However, upon Notch activation by treatment of the NSC cultures with a soluble form of the Notch receptor ligands Delta4 or Jagged1, only Hes3 exhibits changes in its expression (mRNA levels increases within 30 min, peaking at 1 h).

It is quite likely, therefore, that several Hes/Hey family members may promote distinct functions in NSCs, or they may cooperate with others. For example, combined deletions of Hes1, Hes3, and Hes5 in mice leads to the depletion of progenitor cells (Hatakeyama et al., 2004; Imayoshi and Kageyama, 2011).

**Figure 1:**
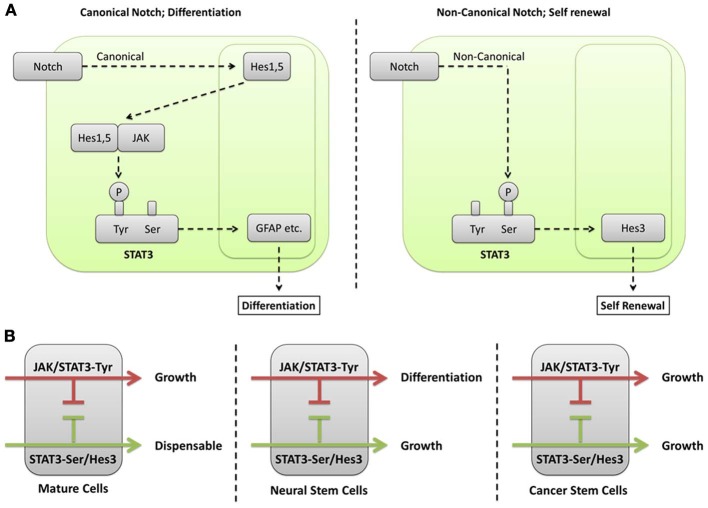
**(A)** Different phosphorylation sites on STAT3 mediate canonical and non-canonical functions of the Notch signaling pathway. In canonical Notch signaling, the cleaved intracellular domain of the Notch receptor translocates to the nucleus where it regulates transcription of target genes, including Hes1 and Hes5. Hes1 and Hes5 are transcription factors themselves and can therefore regulate additional target genes downstream of Notch activation. However, they also have cytoplasmic functions where they form complexes with JAK2 promoting its ability to phosphorylate STAT3-Tyr (Kamakura et al., 2004). This modification leads to the induction of differentiation in NSCs (Bonni et al., 1997). A non-canonical branch of Notch signaling leads to STAT3-Ser phosphorylation in the absence of detectable STAT3-Tyr phosphorylation (Androutsellis-Theotokis et al., 2006). This is followed by increased transcription of Hes3. Hes3, in turn, promotes growth through poorly understood mechanisms. A clue as to the function of Hes3 comes from observations that force-expressed Hes3 in NSC cultures increases the expression of sonic hedgehog (Androutsellis-Theotokis et al., 2006), a morphogen that also acts as a mitogen for NSCs (Ahn and Joyner, 2005). **(B)** Different cell types interpret STAT3 and Hes/Hey signaling in specific ways. Many mature cell types (e.g., neurons and astrocytes in the brain), as well as many established cell lines from different tissues utilize the JAK/STAT pathway that leads to STAT3-Tyr phosphorylation for growth. In contrast, STAT3-Ser phosphorylation is only auxiliary to the transcriptional strength of STAT3 and dispensable. NSCs interpret these signals in a different way. The JAK/STAT pathway involving STAT3-Tyr phosphorylation induces their differentiation, preferentially to the astrocytic fate. NSCs can now recruit the STAT3-Ser phosphorylation as a critical regulator of growth. An important mediator of STAT3-Ser action is Hes3. Cancer stem cells (from glioblastoma multiforme) are able to grow utilizing either the JAK-STAT or the STAT3-Ser/Hes3 signaling pathways. The ability of cancer stem cells to grow utilizing distinct signaling pathways blurs the line distinguishing cancer stem cells from more differentiated cancer cells and underlines their ability to evade therapy aimed at disrupting one or a limited set of signaling pathways.

Our understanding of the function of different Hes/Hey genes received a great boost through the realization that different branches of the STAT3 signaling pathway regulate and/or involve distinct members of their family. For example, Hes1 and Hes5 form complexes with JAK2, promoting its ability to phosphorylate STAT3-Tyr (Kamakura et al., 2004). In this way, Hes1 and Hes5 are part of the pro-differentiation program in cultured NSCs, resulting in increased astrocyte generation. This is consistent with the *in vivo* observations showing that Hes1 and Hes5 oppose the acquisition of the neuronal fates. However, the question remains open as to the extent to which the Hes1+/Hes5+ cell population in the brain is undifferentiated (i.e., are these NSCs, or more differentiated intermediates)? Alternatively, the NSC population may be defined as the Hes1+/Hes5+/Hes3+ population. This is consistent with the observations that Hes3 expression in NSC cultures is opposed by JAK activity and supported by various treatments that promote STAT3-Ser phosphorylation (Androutsellis-Theotokis et al., 2006, 2008b, 2009, 2010a,b). In fact, Hes3 can be used as a NSC biomarker *in vitro* because it is expressed in NSCs and lost upon induction of differentiation. *In vivo*, Hes3+ cells are found in the established neurogenic zones (subventricular zone, dentate gyrus) as well as many other areas of the adult brain; these can be microdissected and cultures can be established efficiently, demonstrating the NSC properties of Hes3+ cells (Poser and Androutsellis-Theotokis, 2013). Hes3 being downstream of STAT3 is consistent with the observation that, unlike Hes1 and Hes5, Hes3 is not a direct transcriptional target of Notch (Kageyama et al., 2005). Together, these results establish that activation of pathways resulting in STAT3-Ser phosphorylation has functions distinct from those of simply supporting JAK-STAT (Figure 1A).

## Activating the STAT3-Ser/Hes3 signaling axis

The role of the STAT3-Ser/Hes3 signaling axis in controlling NSC number *in vitro* and *in vivo* makes it a potential pharmacological target in neurodegenerative disease therapy (Kittappa et al., 2012). Several diverse treatments that promote the pathway exhibit similar results on cultured cell number, including ligands of the Notch (Delta4, Jagged1) and Tie2 (Angiopoietin 2) receptors, basic FGF, and insulin (Androutsellis-Theotokis et al., 2006, 2008b, 2009, 2010a,b). *In vivo*, Delta4, Angiopoietin 2, insulin, or a combination of these along with an inhibitor of JAK significantly increase the number of endogenous NSCs, establishing the predictive properties of these culture systems (Androutsellis-Theotokis et al., 2008a; Poser and Androutsellis-Theotokis, 2013). These factors also have known effects on blood vessels with, Angiopoietin 2 promoting angiogenesis whereas Delta4 opposes it (Maisonpierre et al., 1997; Yancopoulos et al., 2000; Noguera-Troise et al., 2006; Hellstrom et al., 2007; Siekmann and Lawson, 2007; Thurston et al., 2007). Together, their antagonistic effects would mitigate any large scale changes in the vasculature that results from their combined therapeutic use, while still yielding a substantial increase in the number of NSCs.

The observation that distinct phosphorylation sites on STAT3 mediate different outputs (Figure 1B) allows for testable predictions and the generation of new potential treatments based upon an understanding of a given cell's state. In the context of NSCs for example, CNTF, through its activation of both the JAK-STAT and phosphoinositide 3' kinase (PI3K) pathways, leads to STAT3-Tyr phosphorylation as well as STAT3-Ser phosphorylation (via activation of mTOR) (Decker and Kovarik, 2000; Yokogami et al., 2000; Levy and Darnell, 2002). JAK-STAT activation by CNTF is responsible for the powerful differentiation effect of CNTF toward the astrocytic fate. Our understanding of STAT3 signaling allowed us to predict that concomitant treatment of NSCs with CNTF and a JAK inhibitor would suppress STAT3-Tyr phosphorylation while maintaining STAT3-Ser phosphorylation, thus converting the culture conditions from pro-differentiation to pro-self-renewal (Androutsellis-Theotokis et al., 2008b).

## Taking advantage of the STAT3-Ser/Hes3 signaling axis

The consequences of activating the STAT3-Ser/Hes3 Signaling Axis *in vivo* revealed new aspects of the regenerative/repair potential of the adult mammalian central nervous system. First, immunohistochemical detection revealed Hes3+ cells throughout the adult rat brain, with the highest densities being found in the subventricular zone lining the lateral ventricles and the dentate gyrus of the hippocampus (Androutsellis-Theotokis et al., 2006, 2009). This is entirely consistent with a putative role of Hes3 as a biomarker of endogenous NSCs/progenitor cells. However, Hes3+ cells were also observed scattered throughout many gray and white matter areas, displaying a characteristic morphology and associating closely with blood vessels. Microdissection of brain and spinal cord areas and subsequent culture at clonal densities demonstrated the growth of Hes3+ cells and their differentiation to neurons, astrocytes, and oligodendrocytes. Robust cultures can be established, taking advantage of the signaling pathway and providing the culture medium with ligands of the Notch and Tie2 receptors (Androutsellis-Theotokis et al., 2008a; Poser and Androutsellis-Theotokis, 2013). Using these cultures, self-renewal and multipotential of Hes3+ cells can be demonstrated (Androutsellis-Theotokis et al., 2009, 2010a,b).

When these factors are injected into the lateral ventricles of rodents, the number of Hes3+ cells in many areas of the brain and spinal cord increases within days, and results in strong behavioral recovery in models of ischemic stroke and Parkinson's disease. (Androutsellis-Theotokis et al., 2006, 2008b, 2009; Masjkur et al., 2012; Pacioni et al., 2012). This benefit occurred in the absence of detectable newly generated and matured neurons, suggesting these cells provide a neuroprotective effect through trophic support rather than outright replacement. Indeed, in the Parkinsonian models, nigrostriatal dopamine neurons that would otherwise have been lost were rescued. These results revealed a mechanism that the adult central nervous system operates for damage repair, coupling vascular signals (e.g., Delta4, Angiopoietin 2) and a widespread multipotent precursor cell to establish a regenerative environment that promotes restoration of function.

As an extension of these observations, carcinogenesis is sometimes viewed as the manifestation of aberrant regenerative mechanisms. The cells that carry the regenerative ability of a tumor, termed cancer stem cells, express genes common to their non-cancerous stem cell counterparts (Hanahan and Weinberg, 2011; Poser et al., 2011). In line with this notion, putative cancer stem cells from the aggressive brain tumor glioblastoma multiforme express Hes3 both in the patient and in culture. Hes3 RNA interference results in increased death in cells isolated from patient biopsies (Park et al., 2013), highlighting another potential therapeutic benefit of targeting the STAT3-Ser/Hes3 Signaling Axis.

## Conclusions

In the 1960s, Joseph Altman's experiments demonstrated, through the use of tritiated thymidine, that the adult mammalian brain contains proliferating cells that can become neurons (Altman and Das, 1965). It took decades to begin appreciating the significance of these findings and eventually a vigorous search started for biomarkers to identify this cellular population. The results of this effort uncovered discrete regions that harbor multipotent progenitor cells, the stem cell niches of the subventricular zone and the dentate gyrus. However, the bias of the search toward proliferating (albeit slowly) cells resulted in the field missing the widespread neural stem cell population that may be largely quiescent, possibly not even being able to generate new neurons in their microenvironment *in vivo*, but nevertheless, are just as critical to the maintenance of the brain as we age, or recover from injury. The reason for the existence of these “parenchymal” stem cells is not clear but it is conceivable that they are the result of an evolutionary adaptation toward brain tissue rigidity, as a means of storing information and lessons learned as the extraordinary regenerative plasticity that lower animals exhibit may be incompatible with long-term memory storage. These cells may have kept some of their progenitor character (e.g., some biomarkers) but have adapted from direct cell replacement to modulating the microenvironment, thus playing a neuroprotective role. In this way, these cells can still contribute to tissue repair without interfering with information storage. This may also be an evolutionary adaptation because as the mammalian brain has grown large, the challenge of new neurons efficiently recapitulating lost neuronal connections has become extremely daunting. This problem is encountered by efforts to replace lost neurons using approaches that involve the grafting of exogenous NSCs or fetal neuronal tissue. Just like placing a bunch of cable bits in a broken computer does not guarantee that the computer will work again, there is still work to be done before we understand how to effectively integrate a graft bolus into the appropriate brain circuitry to facilitate recovery. Until then, boosting natural, innate repair mechanisms, such as those involving the STAT3-Ser/Hes3 Signaling Axis, represents a sound strategy that both seems to work and addresses disease progression.

### Conflict of interest statement

The authors declare that the research was conducted in the absence of any commercial or financial relationships that could be construed as a potential conflict of interest.
